# Budesonide suppresses pulmonary antibacterial host defense by down-regulating cathelicidin-related antimicrobial peptide in allergic inflammation mice and in lung epithelial cells

**DOI:** 10.1186/1471-2172-14-7

**Published:** 2013-02-06

**Authors:** Peng Wang, Xiaoyun Wang, Xiaoqiong Yang, Zhigang Liu, Min Wu, Guoping Li

**Affiliations:** 1 Inflammations & Allergic Diseases Research Unit, Affiliated Hospital of Luzhou Medical College, Luzhou, Sichuan, 646000, China; 2 Bao Ji Central Hospital, Bao Ji, Shan Xi, 721008, China; 3 Department of Respiratory Disease, Affiliated Hospital of Luzhou Medical College, Luzhou, 646000, China; 4 State Key Laboratory of Respiratory Disease for Allergy at Shenzhen University, School of Medicine, Shenzhen University, Nanhai Ave 3688, Shenzhen, Guangdong, 518060, PR China; 5 Department of Biochemistry and Molecular Biology, University of North Dakota, 501 N Columbia Rd, EJRF Building Room 2726, Grand Forks, North Dakota, 58203-9037, USA

**Keywords:** Allergic airway inflammation, Antibacterial host defense, Cathelicidin, Budesonide

## Abstract

**Background:**

Glucocorticoids are widely regarded as the most effective treatment for asthma. However, the direct impact of glucocorticoids on the innate immune system and antibacterial host defense during asthma remain unclear. Understanding the mechanisms underlying this process is critical to the clinical application of glucocorticoids for asthma therapy. After sensitization and challenge with ovalbumin (OVA), BALB/c mice were treated with inhaled budesonide and infected with *Pseudomonas aeruginosa* (*P*. *aeruginosa*). The number of viable bacteria in enflamed lungs was evaluated, and levels of interleukin-4 (IL-4) and interferon-γ (IFN-γ) in serum were measured. A lung epithelial cell line was pretreated with budesonide. Levels of cathelicidin-related antimicrobial peptide (CRAMP) were measured by immunohistochemistry and western blot analysis. Intracellular bacteria were observed in lung epithelial cells.

**Results:**

Inhaled budesonide enhanced lung infection in allergic mice exposed to *P*. *aeruginosa* and increased the number of viable bacteria in lung tissue. Higher levels of IL-4 and lower levels of IFN-γ were observed in the serum. Budesonide decreased the expression of CRAMP, increased the number of internalized *P*. *aeruginosa* in OVA-challenged mice and in lung epithelial cell lines. These data indicate that inhaled budesonide can suppress pulmonary antibacterial host defense by down-regulating CRAMP in allergic inflammation mice and in cells *in vitro*.

**Conclusions:**

Inhaled budesonide suppressed pulmonary antibacterial host defense in an asthmatic mouse model and in lung epithelium cells *in vitro*. This effect was dependent on the down-regulation of CRAMP.

## Background

Asthma is a chronic inflammatory disorder characterized by airway inflammation and airway hyperresponsiveness. Disease is mediated by increased levels of T-helper 2 (Th2) cytokines, interleukin (IL)-4, IL-5, and IL-13 and elevated serum IgE [1]. The lungs are always exposed to the environment and its microbial components. Infections of the respiratory tract are the most common diseases. Epidemiological investigations have indicated that allergic asthma is a risk factor for pulmonary infection [2,3]. Patients with atopic asthma also develop more infections than non-atopic individuals.

The innate immune system is the first line of host defense. It is responsible for the immediate recognition and regulation of microbial invasion. The innate immune system consists of a range of pre-existing, rapidly mobilized host cellular defenses, including neutrophils, macrophages, epithelial cells, mast cells, eosinophils, and natural killer cells [4]. Airway epithelial cells are an active part of the innate pulmonary immune system and are capable of recognizing microorganisms and secreting host defense molecules, including antimicrobial and antiviral proteins [5]. Antimicrobial peptides (AMPs) have significant antimicrobial activity. Cathelicidins are expressed in bone-marrow-derived and epithelial cells, and have antimicrobial action against bacteria, viruses, and fungi [6]. Low levels of cathelicidin expression can increase susceptibility to infections [7,8]. Th2 cytokines can inhibit antimicrobial host defense in individuals with allergic diseases, and treatment for atopic dermatitis with corticosteroids can cause a strong reduction in AMP levels in both human skin and essential-fatty-acid-deficient (EFAD) mice [9,10].

Glucocorticoids, which are widely regarded as the most effective treatment for asthma, can inhibit the production of most cytokines [11]. In chronic obstructive pulmonary disease, inhaled corticosteroids can increase the risk of pneumonia [12]. Although glucocorticoids have a direct impact on the innate immune system, their effect on asthma remains unclear. To determine the effect of budesonide on antibacterial host defense and allergic airway inflammation, mice and a murine lung epithelial cell line (MLE-12) were treated with budesonide and infected with *Pseudomonas aeruginosa*. Our results show that inhaled budesonide suppressed pulmonary antibacterial host defense and this effect depended on the down-regulation of cathelicidin-related antimicrobial peptide (CRAMP).

## Results

### Effects of budesonide on lung inflammation upon exposure to *P*. *aeruginosa*

**Figure 1:**
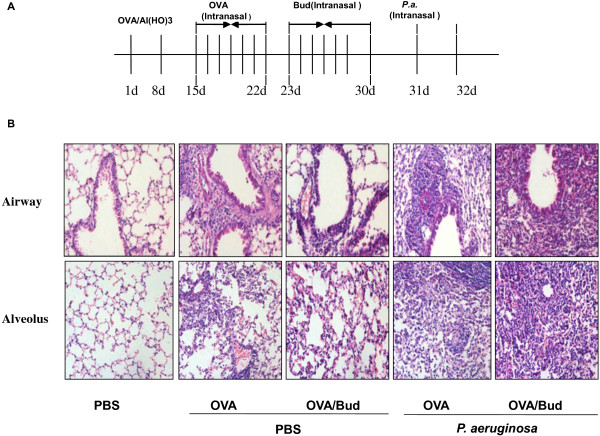
**Effects of budesonide on lung infection in mice exposed to *P. aeruginosa. *A**) Animal model. Mice were sensitized by intraperitoneal injection with ovalbumin (OVA) in aluminum hydroxide on days 1 and 8. From days 15 through 22, mice were intranasally challenged with OVA. OVA-challenged mice were treated with inhaled budesonide on days 23 through 30. On day 31, budesonide-treated mice were infected intranasally with 1 x 10^7^ CFU ml^-1^*P*. *aeruginosa*. Mice were killed after 24 h of *P*. *aeruginosa* infection. **B**) Lung samples were stained with hematoxylin and eosin (HE) (original magnification ×200). OVA/Bud mice had less extensive infiltration of inflammatory cells in the airways than OVA/No mice. However, OVA/Bud/P.a mice developed more inflammation and cellular infiltration than OVA/P.a mice.

Inhaled budesonide inhibits allergic airway inflammation, but the mechanisms involved in increased risk of lung infection in asthmatic patients exposed to bacteria remains unclear. Twenty-four hours after the last dose of inhaled budesonide, histological analysis showed there was less inflammatory infiltration in OVA-challenged mice treated with budesonide (OVA/Bud) than in untreated OVA-challenged mice (OVA/No) (Figure 1B). The cellular infiltration scores of OVA/Bud mice were lower than those of OVA/No mice (Table 1).

**Table 1:** The extent of lung inflammation were analyzed by microscopical histopathologic scoring 24 h after intrartracheal challenge with P. aeruginosa

**Treatment group**	**No. of mice with the following lung pathology score/total no. of mice (%)**			**Lungabscess incidence**
	**1+2**	**3**	**4**	**(%)**
Control	9/10(90)	1/10(10)	0	0
OVA	3/10(30)	6/10(60)	1/10(10)	10
OVA/P.a	0	7/10(70)	3/10(10)	30
ONA/BUD	7/10(70)	3/10(30)	0	0
OVA/BUD/P.a	0	3/10(30)	7/10(60)**	70**

The cellular infiltration scoring of OVA/Bud mice was lower than that in OVA. The scoring of OVA/Bud/P. a mice was higher than that in OVA/P. a *p<0.001 compared to OVA; **p<0.001 compared to OVA/.a.

This confirmed that inhaled budesonide inhibited allergic airway inflammation in OVA/Bud mice. However, we observed different results when mice were infected with *P*. *aeruginosa*. After treatment with inhaled budesonide, diffuse inflammatory cell infiltration and lung abscesses were observed in OVA-challenged mice exposed to *P*. *aeruginosa* (OVA/Bud/P.a). The cellular infiltration scores of OVA/Bud/P.a mice were higher than those for OVA-challenged mice exposed to *P*. *aeruginosa* (OVA/P.a). This indicated that *P*. *aeruginosa* infection increased lung inflammation in OVA-challenged mice treated with inhaled budesonide compared with untreated OVA-challenged mice.

### Effects of budesonide on bacterial levels in OVA-challenged mice exposed to *P*. *aeruginosa*

**Figure 2:**
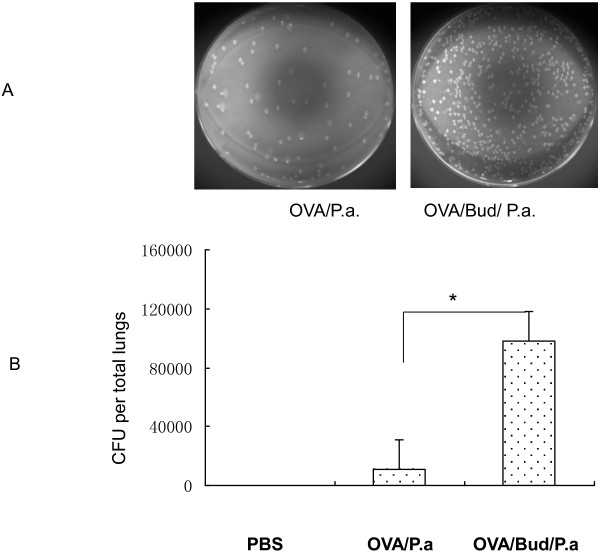
**Measurement of CFUs in lung homogenates by quantitative culture of serial dilutions on blood agar plates.** Figure 2**A** represents the bacterial colonies on blood agar plates between OVA/P.a mice and OVA/Bud/P.a mice observed using a Kodak Image 4000MM. Figure 2**B** represents CFUs, shown in column configuration. There were significantly more CFUs in OVA/Bud/P.a mice than in OVA/P.a mice (*P<0.05). Control mice receiving PBS only had no observable CFUs (0 CFU).

To determine whether budesonide can increase the risk of pulmonary infection in asthma patients, bacterial levels in the lungs were determined 24 h after infection of mice with *P*. *aeruginosa*. Higher numbers of bacterial colony forming units (CFU) were observed in OVA/Bud/P.a mice than in OVA/P.a mice (*P<*0*.*05*) (Figure 2). The number of CFU in control mice, which received phosphate-buffered saline (PBS) instead of active bacteria, was zero. These data show that inhaled budesonide can reduce the clearance of *P*. *aeruginosa* and increase pulmonary infection in OVA-challenged mice.

### Effects of budesonide on IL-4 in OVA-challenged mice exposed to *P*. *aeruginosa*

**Figure 3:**
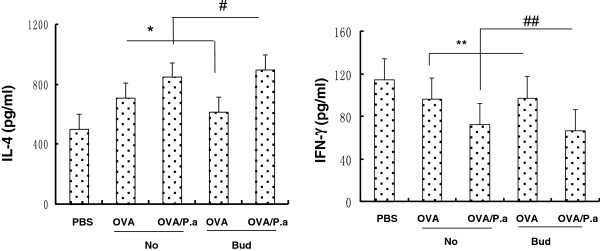
**Serum levels of IL-4 and IFN-γ.** Cytokines in serum were determined by ELISA after 24 h of *P*. *aeruginosa* infection. IL-4 was lower in OVA/Bud mice than in OVA/No mice (*P<0.01). IL-4 in OVA/Bud/P.a mice was higher than in OVA/P.a mice (^#^*P*<0.01). IFN-γ in OVA/Bud mice was not significantly different from that of OVA/No mice (**P>0.05). IFN-γ in OVA/Bud/P.a mice was not significantly different from that of OVA/P.a mice (^##^*P*>0.05). IL-4 in PBS control mice was lower than in all other mice (OVA/No, OVA/Bud and OVA/Bud/P.a). IFN-γ in OVA/No, OVA/Bud, and OVA/Bud/P.a mice was lower than in PBS control mice (*P*<*0*.*01*).

Levels of IL-4 and interferon (IFN)-γ in the serum of mice exposed to *P*. *aeruginosa* were detected to determine the relationship between inhaled budesonide and Th1/Th2 immunoreactions for antibacterial host defense during asthma. The levels of IL-4 in serum were lower in OVA/Bud mice compared with those in OVA/No mice (*P<0.01). However, the levels of IL-4 in OVA/Bud/P.a mice were higher than those in OVA/P.a mice (^#^*P*<*0*.*01*). IL-4 levels were lower in PBS control mice (PBS) than in other mice (OVA/No, OVA/Bud and OVA/Bud/P.a mice) (Figure 3). The level of IFN-γ in OVA/Bud mice was not significantly different from that of OVA/No mice (**P>*0*.*05*). There was also no significant difference between levels of IFN-γ in OVA/Bud/P.a mice and OVA/P.a mice (^##^*P*>*0*.*05*). IFN-γ levels were lower in OVA/No, OVA/Bud, and OVA/Bud/P.a mice compared with those in PBS control mice (PBS) (*P*<*0*.*01*) (Figure 3). These data demonstrated that inhaled budesonide could decrease IL-4 levels in OVA-challenged mice and that *P*. *aeruginosa* infection could increase IL-4 levels in OVA/Bud/P.a mice.

### Effects of budesonide on CRAMP in OVA-challenged mice exposed to *P*. *aeruginosa*

**Figure 4:**
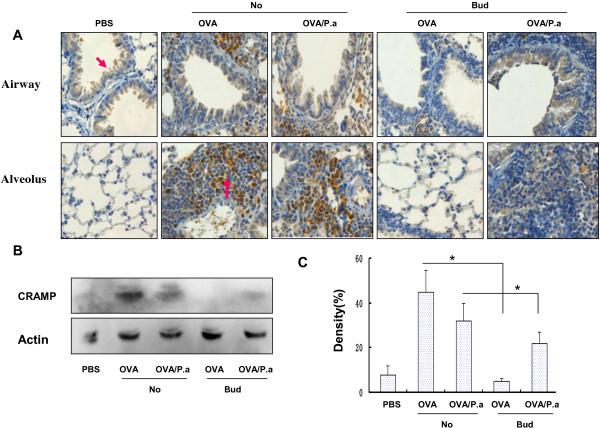
**Expression of CRAMP in lungs by immunohistochemistry and western blot analysis. A**) CRAMP expression and localization in lung tissues was determined by immunostaining (original magnification ×200). **B**) CRAMP expression in lung tissue was determined by western blot analysis. **C**) Densitometry analysis of CRAMP in lung tissues. Both immunohistochemistry and western blot analysis showed that expression of CRAMP in OVA/Bud/P.a mice was markedly lower than in OVA/P.a mice. CRAMP expression in PBS control mice was significantly lower than in OVA/No, OVA/P.a and OVA/Bud/P.a mice.

Airway epithelial cells can be induced to secrete AMPs. Whether inhaled budesonide can inhibit AMPs remains unclear. In the present study, AMP was expressed in epithelial cells of normal lungs and in epithelial cells and inflammatory cells in OVA-challenged mouse lung tissues (Figure 4A). CRAMP expression was significantly lower in PBS control mice (PBS) than in OVA/No, OVA/P.a, and OVA/Bud/P.a mice. This indicated that OVA and *P*. *aeruginosa* caused lung tissues to secrete AMPs. However, CRAMP expression was significantly lower in OVA/Bud mice than in OVA-challenged mice without inhaled budesonide (OVA/No mice). CRAMP expression was significantly lower in OVA/Bud/P.a mice than in OVA/P.a mice (Figure 4B). Thus, inhaled budesonide reduced the production of CRAMP during the antibacterial immune response to asthma.

### Effects of budesonide on the antibacterial host defense of lung epithelial cells

**Figure 5:**
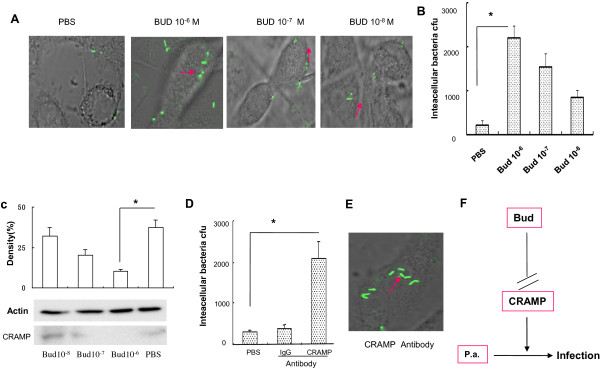
***In vitro *effects of budesonide on the antibacterial host defense of airway epithelium cells. A**) MLE-12 cells were pretreated with budesonide, infected with GFP-labeled *P*. *aeruginosa* and observed by confocal microscopy (original magnification ×400). **B**) MLE-12 cells were lysed and plated to confirm the presence of intracellular bacteria. Bacteria were counted using drop plates. **C**) CRAMP expression in MLE-12 cells was determined by western blot analysis. **D**) MLE-12 cells were pretreated with CRAMP neutralizing antibody. MLE-12 cells were lysed and plated to confirm the presence of intracellular bacteria. Bacteria were counted using drop plates. **E**) MLE-12 cells were pretreated with CRAMP neutralizing antibody. GFP-labeled *P*. *aeruginosa* were observed by confocal microscopy (original magnification ×400). **F**) Effects of budesonide on respiratory infection due to *P*. *aeruginosa.*

Allergic airway inflammation suppresses the innate antimicrobial host defense [9]. However, whether inhaled budesonide can attenuate the antibacterial host defense in airway epithelial cells remains unclear. In our present studies, MLE-12 cells were pretreated with budesonide. We observed that budesonide increased the levels of internalized GFP-labeled *P*. *aeruginosa* in MLE-12 cells (Figure 5A). Total bacterial CFUs were significantly higher in MLE-12 cells exposed to budesonide than in those exposed to PBS. The effect of budesonide on bacterial CFUs in MLE-12 cells was dose-dependent (Figure 5B). High doses of budesonide (10^-6^ and 10^-7^ M) induced significantly lower levels of CRAMP compared with PBS or 10^-8^ M budesonide (*P*<0.01) (Figure 5C). Thus, the role of CRAMP in antibacterial host defense may be interesting. Incubation of MLE-12 cells with CRAMP-neutralizing antibody induced significantly higher levels of internalized GFP-labeled *P*. *aeruginosa* and bacterial CFUs than incubation with isotype IgG antibody (Figure 5D, E). This indicated that budesonide might attenuate the antibacterial host defense of airway epithelium cells by down-regulating CRAMP (Figure 5F).

## Discussion

Allergic asthma is a complex chronic inflammatory airway disease in which many immune cells such as mast cells, eosinophils, T lymphocytes, macrophages, neutrophils, and epithelial cells and cellular elements play different roles. The Th2 hypothesis for asthma was first proposed by Mosmann in 1989 [13]. He identified two different subtypes of T helper cells in mice namely Th1 and Th2 [14] that produced a variety of cytokines and were reciprocally inhibitory. Th1 cells produce IFN-γ, IL-2 and IL-12, which activate mechanisms important for defense against viruses and bacteria [13]. Th2 cells produce cytokines (IL-4, IL-5, IL-6, IL-9, and IL-13), which are important in allergic inflammation and defense against parasites. The Th2 hypothesis of asthma suggests that an imbalance in Th1/Th2 immunity plays an important role in the pathogenesis of allergic asthma [15]. Inhaled corticosteroids (ICS) are recommended as a first-line treatment for asthma by international guidelines. However, a study conducted by Ma et al. showed that the early application of glucocorticoids was a risk factor for human enterovirus 71(HEV71) infection [16]. Recent studies have also shown that topical glucocorticoids compromise the barrier function of normal skin, especially during atopic dermatitis [17,18]. Jamieson et al. showed that a sustained increase in serum glucocorticoid levels in mice with influenza suppressed the systemic antibacterial innate immune response [19]. In the present study, inhaled budesonide decreased the extent of inflammation and cellular infiltration in the airways and the level of IL-4 in OVA-challenged mice. Because allergic airway tissues contain cytokines that promote bacterial infection and colonization in asthma and other lung diseases, Th2 cytokines may be relevant to infection in asthma patients [20]. However, OVA-challenged mice treated with inhaled budesonide and exposed to *P*. *aeruginosa* were characterized by the extensive infiltration of numerous inflammatory cells around bronchioles, alveoli, and blood vessels. This indicated that inhaled budesonide increased lung inflammation, reduced the clearance of *P*. *aeruginosa*, and increased the severity of pulmonary infection in OVA-challenged mice exposed to *P*. *aeruginosa*. Infection with *P*. *aeruginosa* was associated with increased levels of IL-4 in OVA-challenged mice treated with budesonide. Thus, our study indicated that inhaled budesonide increased lung infection in mice with allergic inflammation exposed to *P*. *aeruginosa*, independent of levels of IL-4.

Airway epithelial cells secrete numerous antimicrobial molecules that are part of the host’s first line of defense against microbial invasion. Antimicrobial products secreted constitutively and/or inducibly by epithelial cells include lysozymes, lactoferrin, defensins, collectins, pentraxins, cathelicidin, secretory leukocyte protease inhibitor (SLPI), and serum amyloid A (SAA) [21]. Defensins and cathelicidins are primary AMP factors expressed in the lung and secreted by airway epithelial cells, macrophages, neutrophils, and other classical host defense cells. Another recent study demonstrated that IL-4 and IL-13 have an inhibitory effect on antimicrobial activity of the airway epithelium, as airway epithelial cells were unable to kill bacteria when incubated with these cytokines [6]. Mice with allergic airway inflammation showed significantly more viable bacteria in their lungs after infection. Th2-based inflammation was also found to suppress host defense and reduce AMP expression in the skin [7,22]. Thus, the adaptive immune system modulates the functions of the innate immune system and allergic inflammatory diseases inhibit antimicrobial host defense.

Inhaled corticosteroids are currently considered the most effective means of reducing airway inflammation, symptoms, and morbidity in patients with asthma. Glucocorticoids were shown to affect the synthesis of antimicrobial peptides in amphibian skin [23], inhibit NF-κB signaling and induce immunosuppression in mammalian cell cultures [24]. Mitchell et al. showed that bronchial biopsy specimens from dexamethasone-treated calves had significantly lower levels of tracheal antimicrobial peptide mRNA expression than untreated controls. Thus, corticosteroids may impair innate pulmonary defenses through the regulation of epithelial antimicrobial peptide expression [25]. Tomita et al. demonstrated that glucocorticoids inhibited the release of β-defensin-2 stimulated by lipopolysaccharide in an airway cell line [26]. Aberg et al. showed that psychological stress and systemic and topical glucocorticoid therapy down-regulated epidermal antimicrobial peptide expression and increased the risk of extracutaneous infection in mice [27]. Roca-Ferrer et al. demonstrated that glucocorticoid treatment could cause a modest (30–40%) inhibition of spontaneous lactoferrin secretion in cultured nasal and bronchial mucosa [28]. However, whether ICS can affect anti-microbial host defense among asthma patients remains unclear. In the present study, inhaled budesonide inhibited the production of CRAMP in the antibacterial immune response of OVA-challenged mice. AMP expression was localized to epithelial cells in normal lung tissues and expressed in epithelial cells and inflammatory cells in lung tissues of allergen-challenged mice. Thus, inhaled budesonide suppressed pulmonary antibacterial host defense in an asthmatic mouse model and was dependent on AMPs.

Inhaled corticosteroids induced candidiasis in clinical trials, but the association between the use of inhaled corticosteroids in patients with asthma and the risk of development of community-acquired pneumonia (CAP) remains controversial.

*P*. *aeruginosa* is the leading pathogenic cause of detrimental chronic lung infections, and is a major determinant of morbidity and mortality. Asthma patients with bronchiectasis are not rare, and their conditions are often exacerbated by *P*. *aeruginosa* status. Airway epithelial cells play a critical role in the orchestration of innate defense and inflammatory responses. *P*. *aeruginosa* can adhere to airway epithelial cells and internalization has been observed [29]. In the present study, budesonide increased the number of internalized *P*. *aeruginosa* organisms in MLE-12 cells *in vitro*. The effect of budesonide on bacteria CFU in MLE-12 cells was dose-dependent. High doses of budesonide significantly decreased CRAMP, which was associated with antibacterial host defense.

## Conclusions

We showed that inhaled budesonide could increase the severity of *P*. *aeruginosa* infection in OVA-challenged mice and attenuate antibacterial host defense in airway epithelial cells by down-regulating CRAMP. These findings may have implications for glucocorticoid treatment of asthma patients.

## Methods

### Materials

BALB/c mice (weight 20 to 25 g and age 6 to 7 weeks) were purchased from the Experiment Animal Center of the Sichuan Academy of Medical Science. They were maintained under standard conditions. All animal experiments were performed in accordance with the guidelines of the affiliated hospital of Luzhou Medical College animal care and use committee. *Pseudomonas aeruginosa* strain *P*. *aeruginosa* 103 was provided by the clinical laboratory of the hospital affiliated with Lu Zhou Medical Collage. MLE-12 was maintained in our lab. *P*. *aeruginosa* labeled with green fluorescent protein (GFP) was provided by Dr. Min Wu of the University of North Dakota (US).

### Sensitization and challenge protocol

BALB/c mice were randomly grouped and sensitized with intraperitoneal injections of 20 μg ovalbumin (OVA) in 50 μl aluminum hydroxide on days 1 and 8. Mice were challenged with intranasal instillation of 20 μg OVA in 50 μl phosphate-buffered saline (PBS) on days 15 through 22 inclusive. Control mice were given 50 μl PBS.

### Acute *P*. *aeruginosa* pneumonia model

OVA-challenged mice were treated with inhaled budesonide (350 μg/kg) for 30 min every day from day 23 through 30 as previously described [30]. Twenty-four hours after the last dose of inhaled budesonide and the last OVA challenge, OVA-challenged mice were anesthetized using diethyl ether. They were then infected intranasally with 1×10^7^ CFUs *P*. *aeruginosa*[31]. Control mice received equivalent doses of PBS. Mice were euthanized 24 hours after infection (Figure 1A).

### Quantitation of bacteria

The lungs were removed, weighed, and homogenized in 10% fetal bovine serum (FBS) in Dulbecco’s modified Eagle’s medium (DMEM), and aliquots were plated on *P*. *aeruginosa*-selective plates. Bacterial colonies were counted after incubation at 37°C for 24 hours, and images were obtained using a Kodak Image Station 4000MM (USA).

### Measurement of IL-4 and IFN-γ in serum

The levels of serum IL-4 and IFN-γ were determined using an ELISA kit (R&D Systems, Minneapolis, MN, USA) in accordance with the manufacturer’s instructions.

### Histopathological analysis

Lung tissue was fixed in 4% paraformaldehyde for 24 hours at room temperature and embedded in paraffin. Five-micrometer serial sections were cut and stained with hematoxylin and eosin (HE) or subjected to immunostaining. They were then observed using light microscopy. The degree of cellular infiltration was scored using previously described methods [32]. Cellular infiltration was scored from 0 to 4 as follows: 0 normal cells; 1 few foci (minimal presence); 2 mild diffuse infiltration; 3 moderate diffuse infiltration; 4 severe diffuse infiltration.

### Immunohistochemistry

The sections were deparaffinized and rehydrated. Endogenous peroxidase activity was blocked with 0.3% hydrogen peroxide and the sections were incubated at room temperature for 10 min. Antigen retrieval was performed with citrate (pH = 6) at 95°C in an aqueous bath. The process lasted 40 min. The sections were incubated with rabbit polyclonal antibody against CRAMP (Abcam, Cambridge, MA, USA) at 37°C for 45 min (1:300). The secondary antibody (Envision™, DAKO, Denmark) was applied (1:500) and incubated at 37°C for 45 min. Finally, the slides were visualized using DAB immunostaining under a light microscope (Leica, Solms, Germany).

### Preparation of *P*. *aeruginosa* for cell experiments

GFP-labeled *P*. *aeruginosa* was incubated overnight in lysogeny broth (LB) culture medium on a shaking platform at 150 rpm. The bacteria were added to 10 ml fresh LB medium and cultured for 1 hour until the mid-log phase. Optical density (OD) was measured at 600 nm. When OD_600nm_ reached 0.3, the bacteria were centrifuged at 8000 ×*g* for 5 minutes at 4°C. Bacteria were washed three times in sterile PBS and the density was adjusted to 0.1 OD (0.1 OD = 1 × 10^8^ cells/ml) in sterile Earle’s salt solution. Cells were infected with *P*. *aeruginosa* at a 10:1 bacteria-cell ratio.

### Cell culture and infection experiments

MLE-12 cells were incubated in 24-well tissue plates at 37°C and 5% CO_2_ in DMEM/F12 culture medium. The cells were pretreated for 48 hours at 37°C with 10^-6^, 10^-7^, and 10^-8^ M budesonide until they reached 85% confluence. Then 10^7^ CFU ml^−1^ GFP-labeled *P*. *aeruginosa* was added to MLE-12 cells. For antibody treatment, MLE-12 cells were grown in serum-free DMEM/F12 culture medium for 24 hours. Then 10 μg/ml of CRAMP antibody or a murine isotype control IgG was added to the cells and incubated for 1 hour. MLE-12 cells were infected with 10^7^ CFU ml^−1^ GFP-labeled *P*. *aeruginosa*. After incubation for 1 hour, the cells were washed with PBS and incubated with fresh medium containing polymyxin 50 μg ml^−1^ to kill extracellular bacteria. After 1 hour, the culture media was removed and samples were plated in LB solid culture medium to confirm that the extracellular bacteria had been killed. The 24-well plates were observed using a Zeiss 510 META confocal microscope (Zeiss, Gottingen, Germany). The cells were then homogenized with PBS and spread on LB plates to determine levels of intracellular bacteria. The plates were cultured at 37°C overnight, and colonies were counted. Duplicates were made for each sample and control [33,34].

### Western blot

Lung tissue and MLE-12 cells were homogenized in lysis buffer (1000 μl RIPA with 10 μl phenylmethanesulfonylfluoride (PMSF), Beyotime, China). To ensure each sample contained equal amounts of protein, a protein assay was performed using a bicinchoninic acid (BCA) concentration measurement kit (Beyotime, China). Twenty micrograms of protein was loaded per lane and then run on 15% sodium dodecyl sulfate-polyacrylamide gel electrophoresis (SDS-PAGE) at 100 V for 90 min. Dissolved proteins were transferred onto a nitrocellulose membrane by electroblotting with an Amersham Ecl Semi-Dry Transfer Unit (15 V for 20 min). Non-specific binding sites were blocked with western confining liquid (Beyotime, China) at 37°C for 1 hour. Rabbit polyclonal antibody against CRAMP (1:500 dilution) was applied to the membranes and incubated overnight at 4°C. The membranes were washed three times in PBS for 10 min each. They were then incubated with HRP-conjugated goat anti-rabbit antibody (1:800 dilution, Beyotime, China) at 37°C for 1.5 hours and washed three times for 10 min each. Chemiluminescent substrate (Beyotime, China) was added to the membrane and exposed strips were evaluated using a Kodak Image Station 4000MM (US).

### Statistical analysis

Data are expressed as mean ± standard error. Statistical analysis was performed using ANOVA (Tukey’s post hoc) or Student’s *t*-test and the level of significance was defined as *P*<0.05 between any two groups. The data were analyzed using SPSS 13.0 software.

## Abbreviations

OVA: Ovalbumin; CRAMP: Cathelicidin-related antimicrobial peptide; AMPs: Antimicrobial peptides; MLE: Murine lung epithelial cell; GFP: Green fluorescence protein; ELISA: Enzyme-linked immunosorbent assay; HE: Hematoxylin and eosin; SDS-PAGE: Sodium dodecyl sulfate–polyacrylamide gel electrophoresis; ANOVA: Analysis of variance; OVA/Bud: OVA-challenged mice treated with budesonide; OVA/No: OVA-challenged mice without budesonide treatment; OVA/Bud/P.a: OVA challenged mice treated with budesonide and exposed to *P*. *aeruginosa*; OVA/P.a: OVA-challenged mice exposed to *P*. *aeruginosa* without budesonide treatment.

## Competing interests

The authors declare that they have no competing interests.

## Authors’ contributions

Conceived and designed the study: GL and ZL. Performed the experiment: PW, XW, GL, MW, and XY. Analyzed the data: PW. Contributed reagents and materials: MW. Wrote the paper: PW and XW. All authors read and approved the final manuscript.
